# Response to: ‘Acute flaccid myelitis in low to middle income countries: diagnosis and surveillance’

**DOI:** 10.1093/braincomms/fcae168

**Published:** 2024-05-10

**Authors:** Sam Olum, Charlotte Scolding, Venice Omona, Kansiime Jackson, Neil Scolding

**Affiliations:** Department of Internal Medicine, Gulu Medical School, Gulu University, Gulu, Uganda; St Mary’s Hospital, Lacor, Gulu, Uganda; Department of Internal Medicine, Gulu Medical School, Gulu University, Gulu, Uganda; St Mary’s Hospital, Lacor, Gulu, Uganda; Translational Health Sciences, Faculty of Medicine, University of Bristol, Bristol BS10 5NB, UK; Emergency Medicine Department, Royal United Hospital, Bath BA1 3NG, UK; Department of Internal Medicine, Gulu Medical School, Gulu University, Gulu, Uganda; St Mary’s Hospital, Lacor, Gulu, Uganda; Department of Internal Medicine, Gulu Medical School, Gulu University, Gulu, Uganda; St Mary’s Hospital, Lacor, Gulu, Uganda; Department of Internal Medicine, Gulu Medical School, Gulu University, Gulu, Uganda; St Mary’s Hospital, Lacor, Gulu, Uganda; Translational Health Sciences, Faculty of Medicine, University of Bristol, Bristol BS10 5NB, UK; Department of Neurology, Gloucestershire Hospitals NHS Trust, Cheltenham GL53 7AN, UK

We are grateful to Helfferich and colleagues^1^ for their interest in our work^2^ and for their thoughtful and insightful comments. We particularly agree that two questions concerning acute flaccid myelitis (AFM)—sensory loss and diagnostic criteria—are of especial interest.

Regarding sensory deficits, we agree (and ourselves indicated^2^) that the finding of a sensory level and painless burns in one of our reported cases does under the current criteria^3^ cast doubt on the AFM diagnosis (notwithstanding the difficulty in imagining an alternative cause for the clinical picture, with such persistent flaccidity).

Current diagnostic criteria^3^ do not stipulate that sensory loss absolutely excludes AFM; rather, they stress that sensory deficits ‘might suggest an alternative diagnosis’ while adding that ‘at present, there are no data describing the frequency of [sensory] features in patients with AFM’.^3^

It may be prudent, however, to retain some flexibility on the point, several studies suggesting that sensory changes are in fact not uncommon. Messacar *et al.*^4^ report US Centers for Disease Control and Prevention surveillance documenting sensory involvement in 21% of cases, while Van Haren *et al.*^5^ record sensory loss in 26/59 (44%) of AFM patients—‘10 had deficits in both pain/temperature and fine touch/vibration, and …[a] sensory level was specified or implied in 6 patients’.

Helfferich *et al.*^1^ reasonably make the point that AFM is mainly an anterior horn disease, so that sensory loss would not be expected. However, it may be valuable to rehearse a comparable discussion from some 70 years ago concerning the archetypical AFM, poliomyelitis. Both James Waldo Lance and Fred Plum, among others, reported significant sensory loss in a small minority of poliomyelitis patients, with a clear sensory level in some.^6-9^ Histopathological studies provided a compelling explanation, namely that the extensive nerve cell destruction in the spinal cord grey matter in some cases was associated with a severe inflammatory reaction often extending to the sensory pathways (particularly the dorsal columns).^9,10^ Rather later, detailed neurophysiological studies of polio patients also confirmed the presence of sensory abnormalities.^11^ It is not difficult to imagine similar changes in current cases of AFM.

The poliomyelitis literature is also of interest in indicating the occasional occurrence of second attacks of polio,^12-14^ an atypical feature again suggested historically in two of our cases.

Concerning diagnostic criteria, Helfferich and colleagues^1^ helpfully suggest pragmatic features appropriate to resource-limited settings that might support a clinical diagnosis of AFM, recognizing that ‘in clinical practice, a certain level of uncertainty is not uncommon’. They rightly emphasize, however, that for scientific studies, ranging from exploration of pathogenesis to the ascertainment of incidence and prevalence, it is vital to retain the highest possible levels of diagnostic rigour—which, they aver, must include MRI scanning. They agree, however, that this then excludes much of the resource-limited world.

Perhaps remembering poliomyelitis might be useful here also. A secure diagnosis hardly depended on imaging; rather, clinical features together with, importantly, virological results were more than sufficient. As Helfferich *et al.*^1^ mention, the World Health Organization has established a network of viral laboratories to conduct surveillance for detecting poliomyelitis re-emergence. One such, the Uganda Virus Research Institute, is based in Entebbe. We have recently gained research funding to conduct viral studies, looking for enteroviruses, arboviruses and other viruses previously associated with flaccid paralysis syndromes, in patients with AFM in four different hospitals across Uganda (with MRI scanning in two). Serum, stool and CSF samples and nasopharyngeal swabs can of course be stored cold for delayed testing after transport. Incorporating viral results into the AFM diagnostic criteria in place of MRI scanning—or perhaps better, as an ‘either/or’—might offer the prospect of presenting AFM criteria that are inclusive of resource-limited settings. Confirmation of the diagnosis would be slower, but in the absence of any useful treatment, this is arguably no major disadvantage. We would very much welcome further dialogue and collaboration with AFM authorities in the global north to explore further this important neurological disease.

## Data Availability

Data sharing is not applicable to this article as no new data were created or analysed.
